# The Spleen as a Target to Characterize Immunomodulatory Effects of Down-Stream Processed *Cyberlindnera jadinii* Yeasts in Atlantic Salmon Exposed to a Dietary Soybean Meal Challenge

**DOI:** 10.3389/fimmu.2021.708747

**Published:** 2021-08-20

**Authors:** Byron Morales-Lange, Jeleel Opeyemi Agboola, Jon Øvrum Hansen, Leidy Lagos, Ove Øyås, Luis Mercado, Liv Torunn Mydland, Margareth Øverland

**Affiliations:** ^1^Department of Animal and Aquaculture Sciences, Faculty of Biosciences, Norwegian University of Life Sciences, Ås, Norway; ^2^Faculty of Chemistry, Biotechnology and Food Science, Norwegian University of Life Sciences, Ås, Norway; ^3^Grupo de Marcadores Inmunológicos en Organismos Acuáticos, Pontificia Universidad Católica de Valparaíso, Valparaíso, Chile

**Keywords:** *Salmo salar*, transcriptomics, ELISA, secondary lymphoid organ, inactivated yeast, autolysed yeast

## Abstract

Aquaculture feeds have changed dramatically from being largely based on fishmeal (FM) towards increased use of plant protein sources, which could impact the fish’s immune response. In order to characterize immunomodulatory properties of novel functional ingredients, this study used four diets, one based on FM, a challenging diet with 40% soybean meal (SBM), and two diets containing 40% SBM with 5% of *Cyberlindnera jadinii* yeast exposed to different down-stream processing conditions: heat-inactivated (ICJ) or autolysation (ACJ). The immunomodulatory effects of the diets were analyzed in the spleen of Atlantic salmon after 37 days of feeding, using a transcriptomic evaluation by RNA sequencing (RNA-seq) and the detection of specific immunological markers at the protein level through indirect Enzyme-linked Immunosorbent Assay (indirect ELISA). The results showed that SBM (compared to FM) induced a down-regulation of pathways related to ion binding and transport, along with an increase at the protein level of pro-inflammatory cytokines such as tumor necrosis factor alpha (TNFα) and interferon gamma (IFNγ). On the other hand, while ICJ (compared to FM-group) maintain the inflammatory response associated with SBM, with higher levels of TNFα and IFNγ, and with an upregulation of creatine kinase activity and phosphagen metabolic process, the inclusion of ACJ was able to modulate the response of Atlantic salmon compared to fish fed the SBM-diet by the activation of biological pathways related to endocytosis, Pattern recognition receptor (PPRs)-signal transduction and transporter activity. In addition, ACJ was also able to control the pro-inflammatory profile of SBM, increasing Interleukin 10 (IL-10) levels and decreasing TNFα production, triggering an immune response similar to that of fish fed an FM-based diet. Finally, we suggest that the spleen is a good candidate to characterize the immunomodulatory effects of functional ingredients in Atlantic salmon. Moreover, the inclusion of ACJ in fish diets, with the ability to control inflammatory processes, could be considered in the formulation of sustainable salmon feed.

## Introduction

In aquaculture, the relationship between nutrition and immune system has been recognized as an important part of the fish production process, due to the maintenance of high production densities continuously faces challenges related to multi-stressor conditions such as infectious diseases and suboptimal nutrition (1). In addition, energy and nutrients provided by the feed are essential to maintain an optimal immune function (2). Future growth in aquaculture depends on feed ingredients that are capable of meeting nutritional needs and of improving the overall health of the fish (3).

The dietary composition of the salmon feed has shifted from marine ingredients such as fishmeal towards increased use of plant ingredients (4, 5). However, high inclusion of plant ingredients such as soybean meal, pea proteins and faba bean in diets for Atlantic salmon (*Salmo salar*) can have adverse effects on growth performance and fish health (6–9). In fish, it has already been described that these plant ingredients, due to an imbalanced nutritional composition, content of fiber and antinutritional factors (ANFs) (10, 11), can induce significant changes in gut-microbiome that affect the mucosal immunity, reducing its protective capacity or causing its overreaction, by increasing the secretion of antimicrobial peptides, immunoglobulins and muc-like proteins (3, 12–14). Considering this, solvent extracted soybean meal (SBM) has been used as a dietary challenge to study the impact of alternative ingredients and functional feed components on gut health. SBM has high level of ANFs, which can disrupt intestinal homeostasis and induce inflammation in the distal intestine, commonly referred to as SBM-induced enteritis (SBMIE) (6, 11, 15).

In recent years, novel microbial ingredients (MI), including bacteria and yeast, are gaining increasing interest as replacement for plant-based diets for salmonids (16, 17). Moreover, these ingredients have other properties beyond their nutritional values such as modulators of fish’s immune response (3, 18–22) through components that can be detected as microbial-associated molecular patterns (MAMPs) by pattern recognition receptors (PRRs) in the fish (3, 23, 24).

Furthermore, Grammes et al. (19) reported that the use of MI in feeds could counteract intestinal inflammation in Atlantic salmon. Nevertheless, feeding is a long-term process and its modulation capacity may not only occur locally in the intestine, but also systemically in active immune organs such as the spleen, since immunity has a wide range of cellular and molecular components that can act in an integrated and systemic way. In fish, the spleen has been considered the primordial secondary lymphoid organ with a key role in the antigen presentation processes and lymphocyte activation, promoting humoral immunity, by the cellular coordination of dendritic cells (DC) and with the specific induction of T cell proliferation (25, 26). In addition, in salmonids such as rainbow trout (*Oncorhynchus mykiss*), it has already been described that fish fed functional diets (with the inclusion of *Lentinula edodes*) were able to regulate the acute inflammatory profile in the spleen, reducing possible harmful responses after a LPS-challenge (27). This could be because splenic antigen-presenting cells (APC) would polarize T cells towards regulatory phenotypes, which are important to control the immune responses and in the maintenance of fish homeostasis (28).

Based on this background, the present study proposes the evaluation of the spleen response, as a target organ for the characterization of immunomodulatory effects of down-stream processed *Cyberlindnera Jadinii* in Atlantic salmon exposed to a dietary SBM challenge. To meet this goal, our methodology combines a transcriptomic evaluation by RNA sequencing (RNA-seq) with the specific detection of immunological markers at the protein level by indirect Enzyme-linked Immunosorbent Assay (indirect ELISA). This is in order to increase the knowledge about the modulation of the immune response in Atlantic salmon fed MI.

## Materials And Methods

### Experimental Design

Atlantic salmon with an average starting weight of 5.71 ± 0.06 g were sorted (at an initial stocking density of 3.21 kg/m^3^) into 100 L replicated tanks exposed to a 24 h-light regime and recirculated fresh water (15°C). Oxygen content of the water was measured throughout the experiment and was maintained at an average of 9.5 ± 0.5 mg L^-1^. Moreover, ammonia nitrogen in the recirculating system was kept below the toxic level for the fish, and no mortality or abnormal behavior in any of the fish was recorded in the experimental period.

In each tank, fish were fed for 37 days using one of the four experimental diets: fishmeal diet as a control diet (FM), 40% soybean meal diet as a challenging diet (SBM), and two diets with 40% SBM and 5% inclusion of *C. jadinii* after different downstream processes: heat-inactivated (ICJ) or autolysed (ACJ). *C. jadinii* yeast used in this experiment was produced by fed-batch fermentation using wood sugars as carbon source according to Lapeña et al. (29).

The diet composition was described in **Table 1** and each diet was formulated to meet the nutrient requirement of salmon and contain similar ratio of digestible protein to digestible energy (30). All diets were fed in 20% of excess based on the feed consumption of fish in each tank.

**Table 1:** Formulation and nutritional composition of experimental diets according to Agboola et al. (3).

	FM	SBM	ICJ	ACJ
Fishmeal^a^	433.4	161.4	158.4	158.4
Soybean meal^b^	0	400	400	400
Wheat gluten meal^c^	170	136	111	111
Yeast	–	–	50	50
Potato starch^d^	120	90	68	68
Fish oil^e^	130	130	130	130
Gelatin^f^	60	60	60	60
Cellulose	80	–	–	–
MCP^g^	0	10.0	10.0	10.0
Premix^h^	5.0	5.0	5.0	5.0
L-lysine^i^	–	3.0	3.0	3.0
DL-Methionine^j^	–	3.0	3.0	3.0
Choline chloride^k^	1.5	1.5	1.5	1.5
Yttrium oxide^l^	0.1	0.1	0.1	0.1
***Diet composition (analyzed)***				
Dry matter	924.3	906.5	899.8	914.3
Crude protein	496.6	477.8	474.1	479.4
Crude lipids	191.5	166.5	162.5	171.0
Ash	71.5	60.0	63.5	64.9
Gross Energy (MJ kg^-1^)	21.6	20.9	20.9	21.2
DP : DE^m^	23.0	22.9	22.7	22.6

^a^LT fishmeal, Norsildmel, Egersund, Norway; ^b^Soybean meal, Denofa AS, Fredrikstad, Norway; ^c^Wheat gluten, Amilina AB, Panevezys, Lithuania; ^d^Lygel F 60, Lyckeby Culinar, Fjälkinge, Sweden; ^e^NorSalmOil, Norsildmel, Egersund, Norway; ^f^Rousselot 250 PS, Rousselot SAS, Courbevoie, France; ^g^Monocalcium phosphate, Bolifor MCP-F, Oslo, Norway Yara; ^h^Premix fish, Norsk Mineralnæring AS, Hønefoss, Norway. Per kg feed; Retinol 3150.0 IU, Cholecalciferol 1890.0 IU, α-tocopherol SD 250 mg, Menadione 12.6 mg, Thiamin 18.9 mg, Riboflavin 31.5 mg, d-Ca-Pantothenate 37.8 mg, Niacin 94.5 mg, Biotin 0.315 mg, Cyanocobalamin 0.025 mg, Folic acid 6.3 mg, Pyridoxine 37.8 mg, Ascorbate monophosphate 157.5 g, Cu: CuSulfate 5H_2_O 6.3 mg, Zn: ZnSulfate 151.2 mg, Mn: Mn(II)Sulfate 18.9 mg, I: K-Iodide 3.78 mg, Ca 1.4 g; ^i^L-Lysine CJ Biotech CO., Shenyang, China; ^j^Rhodimet NP99, Adisseo ASA, Antony, France; ^k^Choline chloride, 70% Vegetable, Indukern SA., Spain; ^l^Y_2_O_3_. Metal Rare Earth Limited, Shenzhen, China. ^m^DP : DE, digestible protein: digestible energy ratio. Calculated using internal digestibility values of various ingredients.

FM, fishmeal-based; SBM, Soybean meal-based, ICJ, 40% SBM and 5% of inactivated C. jadinii; ACJ, 40% SBM and 5% of autolyzed C. jadinii (ACJ). Diet formulation and composition are expressed in g kg^-1^ unless otherwise stated.

After the 37-day feeding period, six fish were randomly selected, anesthetized using metacaine at 50 mg L^-1^ and killed with a sharp blow to the head per tank. The individual body weight of each fish was recorded. The final weight per dietary group was 26.01 g ± 0.30 (FM), 24.50 g ± 1.11 (SBM), 24.33 g ± 0.66 (ICJ), 23.53g ± 1.46 (ACJ). No significant differences were detected in the final weight of the fish among dietary groups.

For this study, the spleen of 40 fish was obtained (10 fish per dietary group from duplicated tanks). Then, for each dietary group, six spleen samples were stored frozen in liquid nitrogen (-80°C) until protein extraction and four spleen samples were immediately suspended in RNAlater and stored overnight in the refrigerator, and then kept at -80 °C until total RNA extraction.

The fish experiment was carried out in the Fish Laboratory of Norwegian University of Life Sciences (Ås, Norway) in accordance with the institutional (Permit No. 174) and national regulations for control of live animal experiments in Norway (Norwegian Animal Welfare Law and Norwegian Animal Experimentation Regulations derived from Directive 2010/63/EU).

### RNA-Seq

Total RNA was extracted from sixteen spleen samples (four per dietary group from duplicated tanks), using the RNeasy Mini Kit (Qiagen) following the supplier’s instructions. Then, each RNA sample was quantified using a NanoDrop TM 8000 spectrophotometer (Nanodrop Technologies). Later, RNA integrity was determined using Agilent Bioanalyzer 2100. All samples showed a RNA integrity number (RIN) ≥ 8. Library preparation and RNA-seq were performed by the Norwegian Sequencing Center (UiO, Norway), using TruSeq Stranded mRNA library prep and Illumina HiSeq 4000 System (150 bp paired-end RNA sequencing).

RNA-seq data analysis was performed according to Håkenåsen et al. (31). Raw reads were cleaned by BBDuk (v34.56) to trim/remove low quality reads, adapter sequences and PhiX (Illumina spike-in) using: ktrim = r, k = 23, mink = 11, hdist = 1, tbo, tpe, qtrim = r, trimq = 15, maq = 15, minlen = 36, forcetrimright = 149. Thereafter, cleaned reads were aligned to *Salmo salar* genome ICSASG_v2 (RefSeq assembly accession: GCF_000233375.1) by HISAT (v2.1.0). Fragments mapping were counted using featureCounts (v1.4.6-p1) and differentially expressed genes (DEGs) were estimated between diets using SARTools R package (v1.7.3). Significant DEGs were determined when the adjusted p value (padj) was < 0.05.

To characterize differentially expressed genes, functional classification was performed using Gene Ontology (GO) analysis by g:Profiler (32). To achieve this, *Salmo salar* genome database (Ensembl) and gene IDs (Entrezgene_ACC) from significant DEGs list were used. GO categories (g:SCS threshold 0.05) were displayed in –log_2_(*p*). In addition, EnrichmentMap v3.3 (33) in Cytoscape v3.81 (34) was used with default settings to visualize all diet comparisons in a single network of GO terms.

To further understand gene biological functions, significant DEGs and their expression values were used for Kyoto Encyclopedia of Genes and Genomes (KEGG) Pathway Enrichment Analysis among dietary groups (by clusterProfiler v3.16.1 package in R). Enriched pathways were selected (pvalueCutoff = 0.05) and displayed as -log_2_(*p*). Enrichment maps were obtained using emapplot (Enrichplot package v.1.8.1 in R).

### Detection of Immunological Markers

For the characterization of the immune response in the spleen of Atlantic salmon after a dietary challenge, biomarkers at the protein level were evaluated in six fish samples per dietary group (from duplicated tanks). Each sample was homogenized using metal beads and RIPA lysis buffer with protease inhibitor cocktail (1x). Then, the samples were centrifuged and total proteins were quantified from the supernatants using the Bicinchoninic acid protein assay kit (Pierce). Thereafter, indirect ELISA was performed following Morales-Lange et al. (35). Briefly, each sample was diluted in carbonate buffer (60 mM NaHCO_3_ pH 9.6) and seeded by duplicate in a 96-well plate (Nunc) at 50 ng μL^−1^ (100 μL) for overnight incubation (4°C). Next day, the plates were washed three times with PBS-Tween20 (PBST 0.2%) and incubated with 200 µL of blocking solution (per well) for 2 h at 37°C (Pierce Clear Milk Blocking Buffer 1x). After successive washes with PBST 0.2%, 100 μL of the primary antibody (**Table 2**) was incubated for 90 min at 37°C and later, a secondary antibody diluted 1:5000 (goat anti mouse IgG-HRP or mouse anti rabbit IgG-HRP) was incubated per well during 60 min at 37°C. Chromagen substrate 3,3′,5,5′-tetramethylbenzidine single solution (TMB, Thermofisher) was added (100 μL) and incubated for 20 min at room temperature (in dark). All reactions were stopped with 50 μL of 1 N sulfuric acid and finally the plates were read at 450 nm in a SpectraMax microplate reader. (Molecular Devices).

**Table 2:** Primary antibodies for indirect ELISA.

Marker	Source	Type	Dilution	Reference
CD3	Mouse	Monoclonal	1:400	(36)
CD4	Rabbit	Polyclonal	1:500	(37)
CD83	Rabbit	Polyclonal	1:200	(38)
IFNγ	Mouse	Polyclonal	1:400	(3)
IgD	Mouse	Monoclonal	1:400	(39)
IgM	Mouse	Monoclonal	1:400	(39)
IL-10	Mouse	Polyclonal	1:400	(40)
MHC II	Mouse	Polyclonal	1:400	(38)
TNFα	Mouse	Polyclonal	1:400	(3)
ZBTB46	Mouse	Polyclonal	1:400	**Supplementary Figure 1**

Results from indirect ELISA were expressed in fold change relative to SBM. GraphPad Prism 8 was used to display the data and calculate means, standard deviations, one-way ANOVA and Tukey’s test for multiple comparisons between diets. Furthermore, Corrplot package in R (41) was used to make correlations among diets. All significant differences were determined when p value was <0.05.

## Results

### Transcriptomics

DEGs number per diet comparison showed different patterns among groups (**Table 3**). The highest differentiated gene expression occurred when fish fed FM were compared to those fed SBM (313 down-regulated, 448 up-regulated). A lower number of DEGs were observed in ACJ-group compared with both FM (230 DEGs down-regulated, 163 DEGs up-regulated) and SBM (95 DEGs down-regulated, 51 DEGs up-regulated). Moreover, in ICJ-fed fish, few numbers of DEGs relative to both FM (seven down-regulated, 21 up-regulated) and SBM (21 down-regulated and four up-regulated) were detected.

**Table 3:** Significant differentially expressed genes (DEGs) per diet-comparison.

Diet-comparison	Downregulated	Upregulated
FM|SBM	313	448
ICJ|FM	7	21
ICJ|SBM	21	4
ACJ|FM	230	163
ACJ|SBM	95	51
ACJ|ICJ	4	3

The comparison between the two diets with *C. jadinii* (ACJ and ICJ) only showed four down-regulated and three up-regulated DEGs. Complete list of significant DEGs along with the name of each gene is attached in Supplementary File 2. In addition, RNA-seq raw data is available in Gene Expression Omnibus database (GEO-NCBI: GSE174262).

#### Gene Ontology

**Figure 1:**
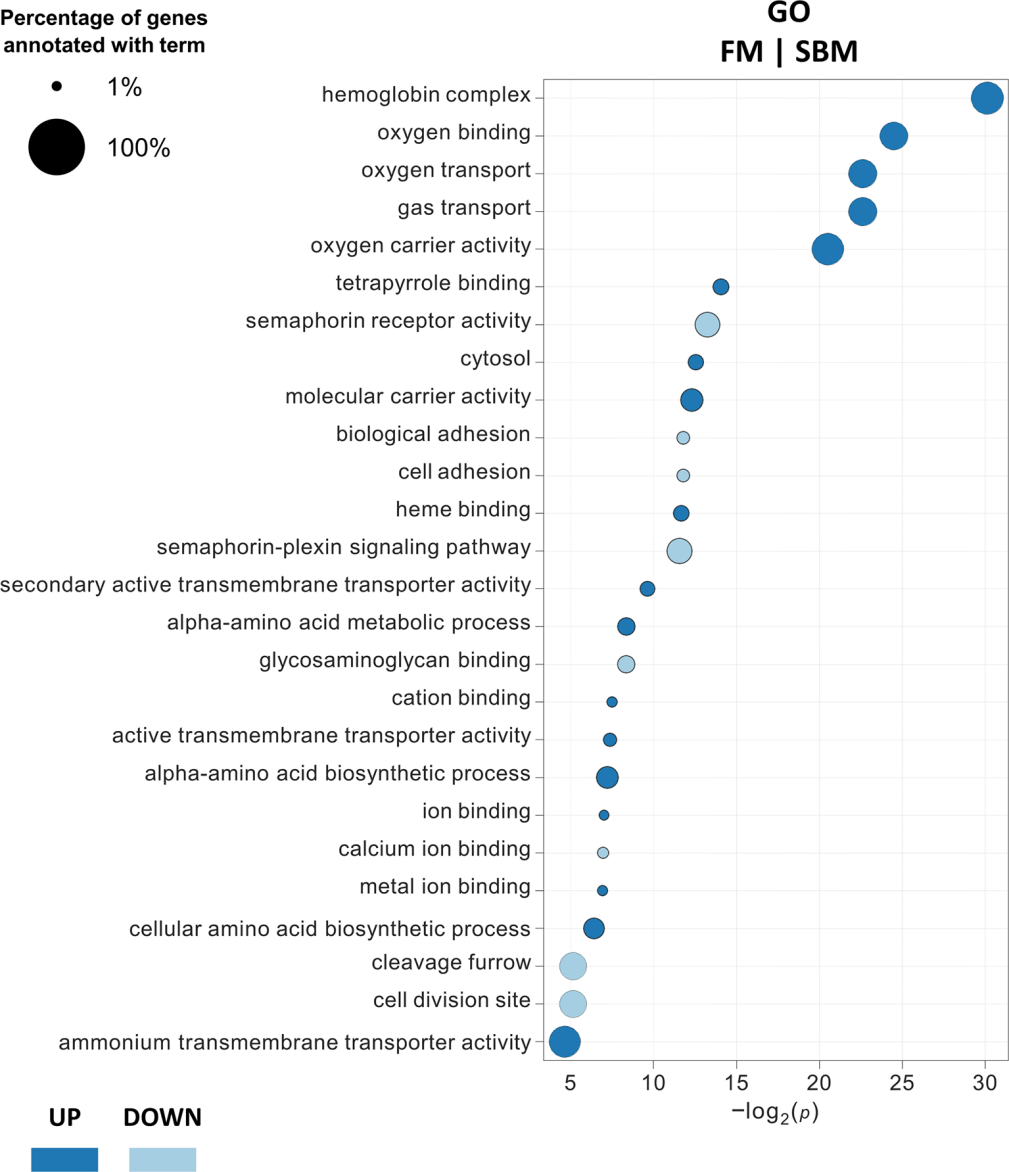
Significantly enriched gene ontology (GO) terms (minGSSize = 3) in spleen of fish fed FM diet compared to SBM. The list is ordered by -log_2_(*p*). Up, up-regulated (in blue); Down, down-regulated (in light blue).

**Figure 2:**
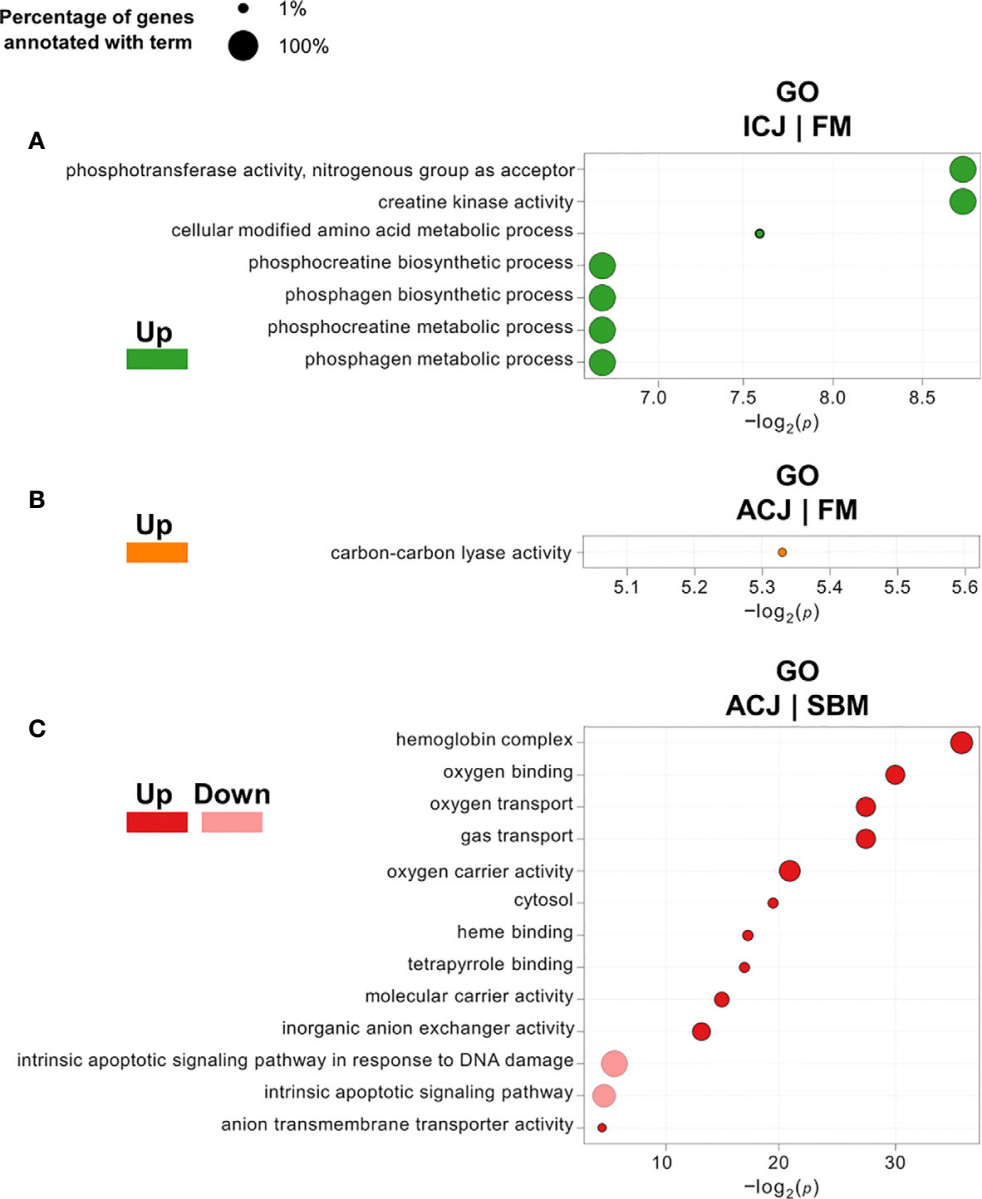
Significantly enriched gene ontology (GO) terms (minGSSize = 3) in spleen of fish fed *C. jadinii* diets compared to control diets (FM and SBM). The list is ordered by -log_2_(*p*). **(A)** GO terms in ICJ compared to FM. Up, up-regulated (in green). **(B)** GO terms in ACJ compared to FM. Up, up-regulated (in orange). **(C)** GO terms in ACJ compared to SBM. Up, up-regulated (in red); Down, down-regulated (in pink).

DEG classification by Gene Ontology using three categories (molecular function, biological processes and cellular components) showed 26 overrepresented GO terms (18 upregulated and 8 downregulated) in FM compared to SBM (FM|SBM, **Figure 1**). The analysis showed that the up-regulated terms in FM were mainly associated with ion binding, transporter and metabolic activity, while down-regulated GO terms were related to semaphorin activity, biological adhesion and cell adhesion. The same analysis comparing ICJ with both control diets showed only overrepresented GO terms (up-regulated) for ICJ compared to FM (ICJ|FM, **Figure 2A**). In this case, ICJ showed seven significant GO terms related to phosphagen metabolic and biosynthetic process. In addition, when comparing ACJ with FM (ACJ|FM, **Figure 2B**), the results showed one GO term up-regulated in ACJ (carbon-carbon lyase activity). On the other hand, the comparison between ACJ and SBM (ACJ|SBM, **Figure 2C**) showed two down-regulated terms (associated to intrinsic apoptotic signaling pathway) and 11 up-regulated terms in ACJ. Interestingly, the up-regulated terms observed in ACJ compared to SBM were similar to when FM was compared to SBM (molecular binding and gas transporter activity). The analysis between ACJ and ICJ did not show differentially significant GO terms.

**Figure 3:**
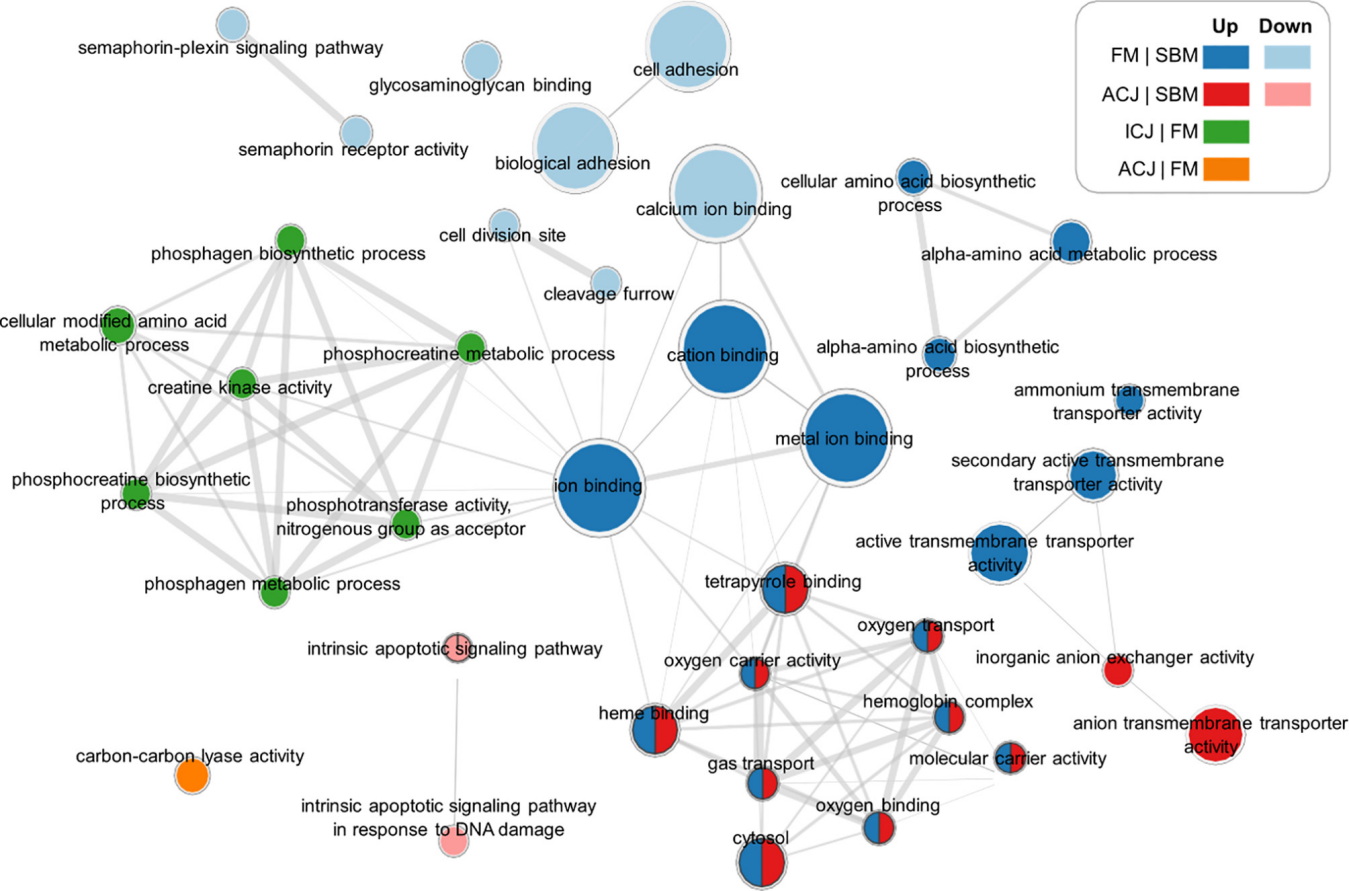
Significant networks of enriched GO terms related to comparisons between different diets. FM|SBM: Up (up-regulated in blue), Down (down-regulated in light blue). ICJ|FM: Up (up-regulated in green). ACJ|FM: Up (up-regulated in orange). ACJ|SBM: Up (up-regulated in red), Down (down-regulated in pink).

By grouping the GO terms detected (from different diet comparisons) in a network (**Figure 3**), we observed that FM and ACJ (compared to SBM) share similarities associated with the up-regulation of tetrapyrrole binding, oxygen transport, oxygen carrier activity, hemoglobin complex, heme binding, gas transport, molecular carrier activity, oxygen binding and cytosol. Furthermore, it was possible to determine that FM compared to SBM (FM|SBM) was related to ICJ compared to FM (ICJ|FM) through ion binding.

#### KEGG Pathway Analysis

**Figure 4:**
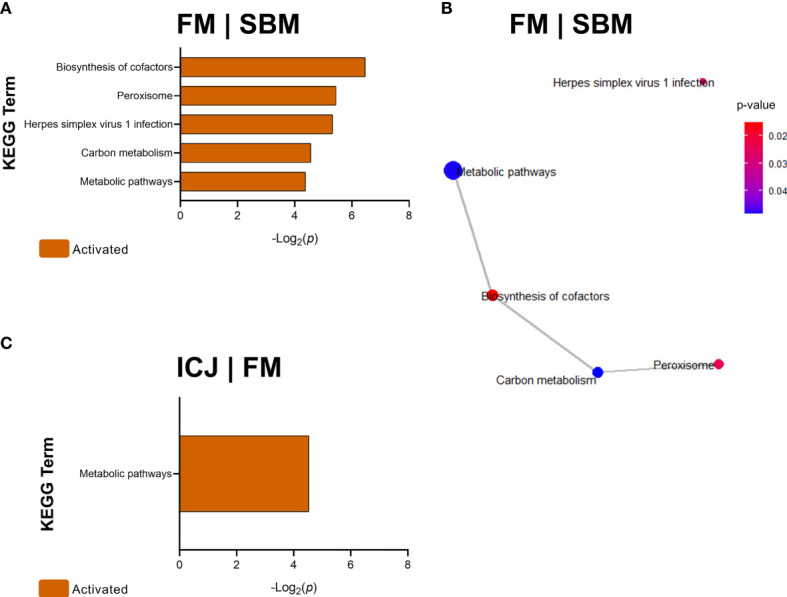
KEGG Pathway Enrichment Analysis of DEGs (minGSSize = 3) from spleen in fish fed FM compared to SBM (FM|SBM) and ICJ compared to FM (ICJ|FM). List ordered by -Log_2_(*p*). **(A)** Significant KEGG terms in FM compared to SBM. **(B)** KEGG network analysis between FM and SBM. **(C)** Significant KEGG terms in ICJ compared to FM.

Pathway analyses according to KEGG showed that DEGs from FM compared with SBM (FM|SBM, **Figure 4A**) were related to five activated KEGG terms: biosynthesis of cofactors, peroxisome, herpes simplex virus 1 infection, carbon metabolism and metabolic pathways. Using these data, the association network showed that four of the five KEGG terms (except herpes simplex virus 1 infection) were related (**Figure 4B**). Moreover, DEGs from ICJ, compared to FM (ICJ|FM, **Figure 4C**), showed only a significant activation of metabolic pathways. The comparison between ICJ and SBM did not show any significant KEGG terms.

**Figure 5:**
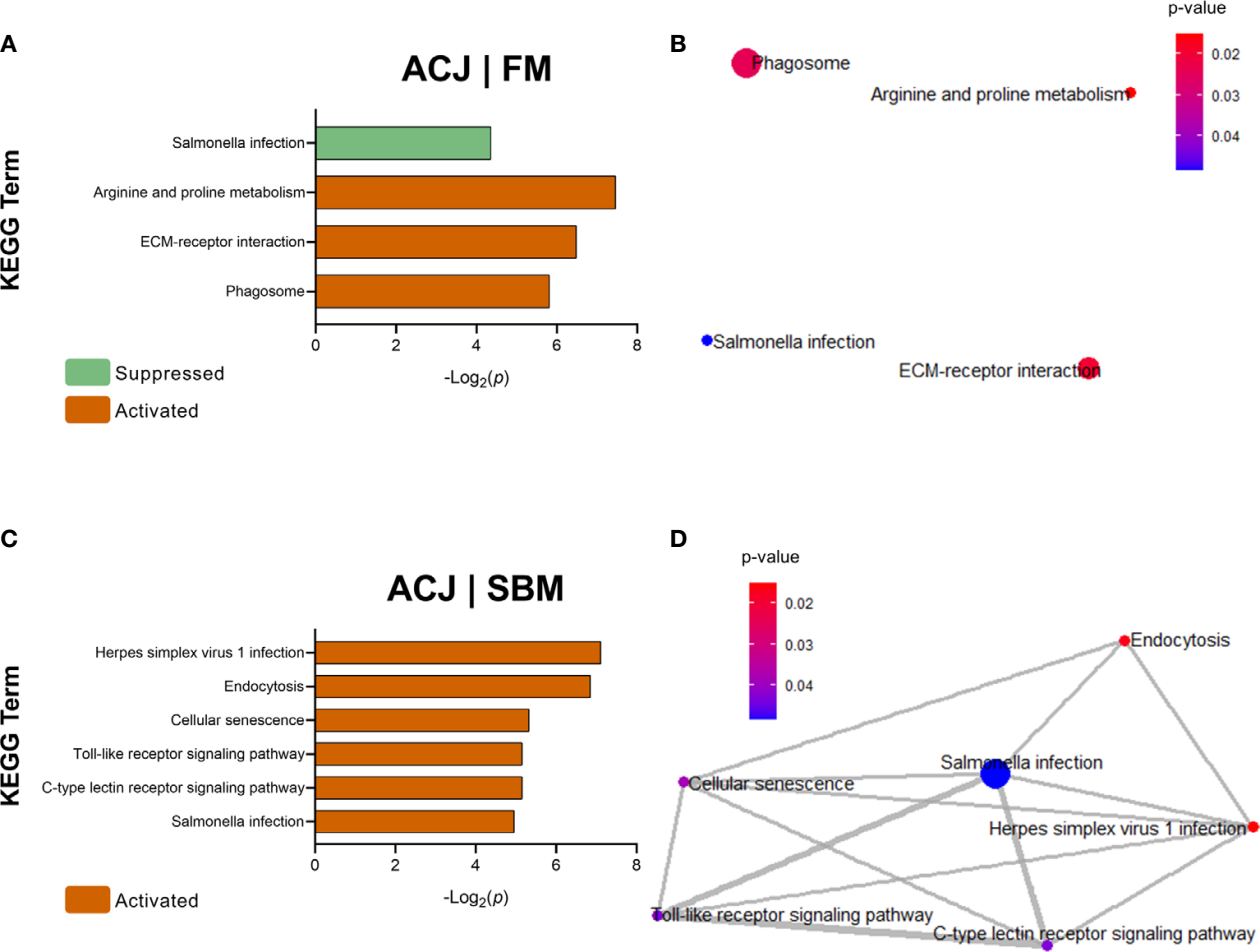
KEGG Pathway Enrichment Analysis of DEGs (minGSSize = 3) from spleen in fish fed ACJ compared to control diets (FM and SBM). In orange: activated pathways, in light green: suppressed pathways. List ordered by -Log_2_(*p*). **(A)** Significant KEGG terms in ACJ compared to FM (ACJ|FM). **(B)** KEGG network analysis between ACJ and FM. **(C)** Significant KEGG terms in ACJ compared to SBM (ACJ|SBM). **(D)** KEGG network analysis between ACJ and SBM.

In **Figure 5A**, DEGs from ACJ compared to FM (ACJ|FM) showed the activation of 3 KEGG terms: arginine and proline metabolism, ECM-receptor interaction and phagosome, and the suppression of salmonella infection-pathway. However, the analysis of interactions from these pathways did not show close relationships (**Figure 5B**).

DEGs comparison between ACJ and SBM (ACJ|SBM) showed six KEGG terms significantly overrepresented in ACJ: herpes simplex virus 1 infection, endocytosis, cellular senescence, Toll-like receptor signalling pathway, C-type lectin receptor signalling pathway and salmonella infection (**Figure 5C**). Enrichment maps using these KEGG terms showed that all pathways were associated in a cluster (**Figure 5D**). Regarding the comparison between ACJ and ICJ, no significant KEGG terms were determined.

### Immunological Markers

**Figure 6:**
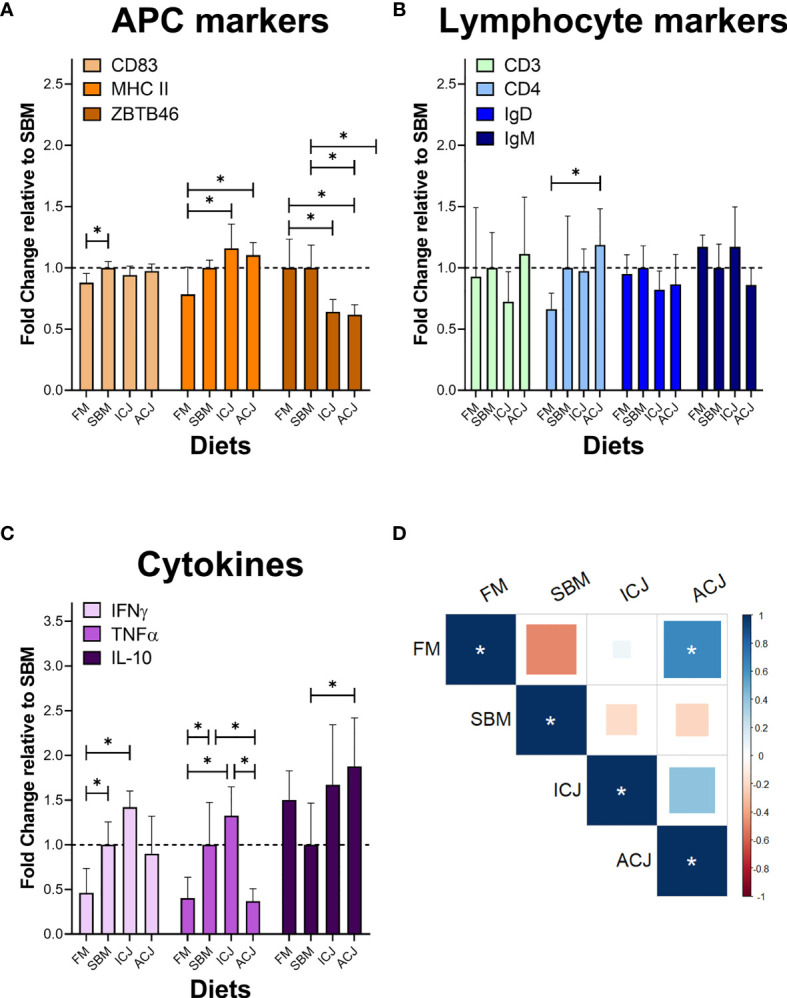
Protein detection of immunological markers in spleen by indirect ELISA. **(A)** Antigen-presenting cell (APC) markers. **(B)** Lymphocyte markers. **(C)** Cytokines. **(D)** Correlation between diets using the data from different immunological markers (Degrees of freedom = 8). In **(A–C)** * shows significant differences among dietary groups (p < 0.05). In **(D)** * significant correlation (p < 0.05).

The detection of immunological markers by indirect ELISA showed lower levels of Cluster of differentiation 83 (CD83) in fish fed FM compared to SBM (0.88-fold, **Figure 6A**). Moreover, an increased production of major histocompatibility complex class II (MHC II) was detected in fish fed both diets with *C. jadinii* inclusion (ICJ= 1.16-fold and ACJ= 1.10-fold, respectively) compared to FM (0.78-fold). In addition, the level of ZBTB46 (Zinc finger and BTB domain-containing protein 46) decreased in fish fed ICJ (0.64-fold) and ACJ (0.62-fold) diets, compared to both FM (1.00-fold) and SBM-fed fish (1.00-fold).

Also in **Figure 6**, only Cluster of differentiation 4 (CD4) was significantly different among diets (**Figure 6B**). It was higher in fish fed ACJ-diet (1.19-fold) compared to FM diet (0.66-fold). Cluster of differentiation 3 (CD3), Immunoglobulin D (IgD) and Immunoglobulin M (IgM) did not show significant differences between groups.

The level of cytokines in SBM-diet group (**Figure 6C**) showed a higher level of interferon gamma (IFNγ: 1.00-fold) and tumor necrosis factor alpha (TNFα: 1.00-fold) compared to FM (0.46-fold and 0.40-fold, respectively). A similar behavior to SBM-fed fish was detected in ICJ-diet group, where both pro-inflammatory cytokines showed an increase in their protein levels (IFNγ= 1.42-fold, TNFα= 1.33-fold) compared to FM. On the other hand, the cytokine values from ACJ-diet group, compared with SBM (**Figure 6C**), showed a reduction of TNFα levels (0.37-fold) and an increase in the availability of interleukin 10 (IL-10: 1.88-fold).

Finally, the correlation of all these immunological markers between the different diets (**Figure 6D**) showed a significant positive correlation among FM and ACJ diets (0.64). Correlations between other diets did not show significant results.

## Discussion

The proposal to consider the spleen as a target candidate for the characterization of immunomodulatory effects of down-stream processed *C. jadinii* in Atlantic salmon has been based on the fact that in fish the spleen has already been considered as the primordial secondary lymphoid organ and it has a central role in the systemic immune response through the coordination of the innate and adaptive immunity by the antigen presentation process (26). In Atlantic salmon, our results at the transcriptional level have shown that this organ also coordinates aspects related to molecular binding, transporter activity, receptor signalling pathways, cellular and metabolic processes, among others, which have already been described as important functions of the spleen in another salmonid specie such as rainbow trout (42).

On the other hand, from the use of different diets, we were able to detect that fish fed SBM diets, compared to FM, showed a down-regulation of GO and KEGG terms linked to ion binding, peroxisome, metabolic and transport-associated pathways. These results are also similar to those reported in intestine of salmon fed SBM diets. In intestine, in addition to inducing an inflammatory profile, SBM decreases barrier functions through the down-regulation of genes associated with iron-binding proteins, detoxification, transport and metabolic processes (43, 44).

The data also showed that the inclusion of SBM in the diets could induced systemic effects in Atlantic salmon that can be detected in the spleen after 37 days of feeding. Furthermore, the results at the protein level showed that SBM also elicits inflammatory responses in the spleen by increasing the production of cytokines such as TNFα and IFNγ, and inducing the availability of CD83. In higher vertebrates, CD83 is a molecule expressed mainly by mature dendritic cells and acts as an immuno-regulator protein by delivering co-stimulatory signals, which can trigger T helper cell-mediated responses that increase TNFα and IFNγ (45, 46). In fish, both cytokines have a key role in the inflammatory process by activating macrophages/phagocytes, thereby enhancing their phagocytic and antimicrobial activity (47).

Regarding the differences between ICJ and ACJ compared to control diets, we hypothesize that the down-stream processing of *C. jadinii* yeasts by autolysis before feed manufacture might explain the observed differences in responses. Different down-stream processes after the yeast is harvested may influence its nutritional value and the accessibility of its cell wall components (16). This could modulate the immune response of Atlantic salmon by exposing fish to different types or amount of MAMP’s from *C. jadinii*, such as β-glucan, mannans, chitin and nucleic acids. Inactivated yeasts have already been described as having smooth surfaces without wrinkles, while autolysed yeasts are partially broken and wrinkled, releasing their intracellular content, exposing bioactive components (3, 22), which can be detected by PRRs from the fish, triggering a different immune response (23, 24).

The autolysis process has been reported to modify the nanomechanical properties of the yeast cell wall (without altering its chemical composition), increasing branching and availability of reactive molecules (48). Furthermore, previous works have described the effect of autolysis on the digestibility of yeast in Atlantic salmon, highlighting the importance of down-stream processing when using MI as protein source in feed production (3, 22).

When comparing ACJ with FM, the data showed activated KEGG terms such as phagosome and arginine and proline metabolism. These pathways are connected to the immune response through processes linked to the ability of cells to engulf solid particles to form internal vesicles (49) and with the maintenance of homeostasis, regulating the antioxidant activity in fish (50, 51). On the other hand, the inclusion of inactivated *C. jadinii* in SBM diets did not show marked differences compared to SBM diets. In fact, it seems to maintain a similar inflammatory profile of SBM compared to FM, with increased levels of TNFα and IFNγ. Regarding TNFα, this trend had already been reported in the intestine of Atlantic salmon fed ICJ (3). It is interesting to note that in fish both molecules (TNFα and IFNγ) would be capable of activating M1 macrophages, due to these cytokines can stimulate phagocytosis and the pro-oxidative process to destroy the potential aggressor (52), which would increase the inflammatory pattern of SBM.

On the contrary, the use of ACJ seems to control and regulate the inflammation caused by SBM. When comparing ACJ to SBM, we observed a similar pattern with the one observed among the comparison of FM with SBM. Both showed GO terms associated with molecular binding and transport. Moreover, ACJ was also able to up-regulate KEGG terms associated to endocytosis and signalling pathways of PRRs. We propose that the increase of IL-10 in ACJ contributed to the reduction of the inflammatory profile associated with SBM, controlling the production of TNFα. In fish, IL-10 is a cytokine that acts as a suppressor and exerts a conserved role in dampening inflammatory responses (47). Furthermore, the cytokine profile in ACJ suggests a modulation of M1/M2 response, which has been reported as conserved in fish (53). While M1 macrophages increases the robustness of the immune response, M2 macrophages act as repair cells, capable of controlling tissue damage caused by both pathogens and the action of the immune system itself, through a phenotype regulated by molecules such as Transforming growth factor beta (TGFβ) and IL-10 (52, 54).

Additionally, Piazzon et al. (55) have reported that the regulatory activities of IL-10 would not only be associated with immunosuppression and M2 phenotype, but could also be related to the maintenance of memory cells over time. However, further studies must be conducted to better understand this relationship. In our work, ACJ was also able to increase CD4 levels compared to FM, nevertheless, its mechanism of action is not possible to explain with the present results. CD4 is the most characterized marker for T lymphocytes, which govern immune responses through specific antigen recognition and subsequent secretion of effector and regulatory cytokines (37). In rainbow trout, it has been described that cross-talk between activated splenocytes can induce an increase in Forkhead box P3 (FOXP3) (28), which is the transcriptional factor associated with polarization of naive T cells to Treg (56).

The positive correlation observed between FM and ACJ suggest a proportional immune response in these diets, which has also been described in the gut of Atlantic salmon (3). Moreover, in rainbow trout, the inclusion of β-glucans derived from fungi (*Lentinula edodes*) was able to control the acute inflammatory response in the spleen, reducing potential harmful responses for the fish (27).

It is also interesting that fish fed diets with *C. jadinii* showed higher levels of MHC II compared to the FM diet, in addition to a lower level of ZBTB46 when compare to the FM and SBM diets. MHC II is a protein involved in the antigen-presentation of peptides derived from exogenous proteins to CD4^+^ T-cells (57). On the other hand, ZBTB46 is a transcriptional factor that inhibits the maturation of APCs in higher vertebrates (58). In salmonids, this molecule has been described in rainbow trout (59). Furthermore, in Atlantic salmon, the modulation of ZBTB46 has been reported in spleen-APCs induced with IFNγ (38). Considering this background, the results in this study suggest an activation of APCs. However, in fish, APCs are still poorly described, and their detection and characterization should be studied deeper in future works to understand their role in the modulation of the immune response by functional diets. Despite this, we propose that the differential activation of APCs in diets with *C. jadinii* compared to SBM (with higher level of CD83, but without other modulated APC markers) would be due to leukocytes from which APCs progress are not a homogeneous subpopulation (38). Moreover, in mammals, APCs could be functional at different stages of maturity, depending on the cytokine environment in which they are found (60, 61).

In summary, we recommend the spleen as a target organ for characterization of immunomodulatory effects of down-stream processed *C. jadinii* in Atlantic salmon exposed to a dietary SBM challenge. Furthermore, our findings contribute to establish a baseline for the study of other novel ingredients that are capable of regulating the immune system of fish, without compromising nutritional parameters. The results from this study suggest that autolysis should be considered when formulating salmon feeds with *C. jadinii* as a functional ingredient with the ability to regulate inflammatory processes.

## Data Availability Statement

The datasets presented in this study can be found in online repositories. The names of the repository/repositories and accession number(s) can be found in the article/**Supplementary Material**.

## Ethics Statement

The animal study was reviewed and approved by Norwegian University of Life Sciences in accordance with the institutional and national regulations for control of live animal experiments in Norway.

## Author Contributions

The study was conceived by BM-L and JOA with key inputs from JØH, LL, and MØ. The experiments and data analysis were performed by BM-L, JOA, and OØ. LM, and LL were in charge of the production and obtaining of antibodies used in this study. The funds for this investigation were acquired by LL, LTM and MØ. BM-L drafted the manuscript with substantial contributions from all other authors. All authors contributed to the article and approved the submitted version.

## Funding

This study was funded by Foods of Norway a Centre for Research-based Innovation (237841/030) and Trained immunity and nutritional programming for resilient salmon (RCN 294821).

## Conflict of Interest

The authors declare that the research was conducted in the absence of any commercial or financial relationships that could be construed as a potential conflict of interest.

## Publisher’s Note

All claims expressed in this article are solely those of the authors and do not necessarily represent those of their affiliated organizations, or those of the publisher, the editors and the reviewers. Any product that may be evaluated in this article, or claim that may be made by its manufacturer, is not guaranteed or endorsed by the publisher.
