# Supplementary material for: The Spleen as a Target to Characterize Immunomodulatory Effects of Down-Stream Processed Cyberlindnera jadinii Yeasts in Atlantic Salmon Exposed to a Dietary Soybean Meal Challenge
Source: Front Immunol. 2021 Aug 20;12:708747. doi: 10.3389/fimmu.2021.708747 (PMC8417602; doi:10.3389/fimmu.2021.708747)
Supplement: Supplementary file 1 [file DataSheet_1.docx]

Supplementary Material

## Supplementary Figures


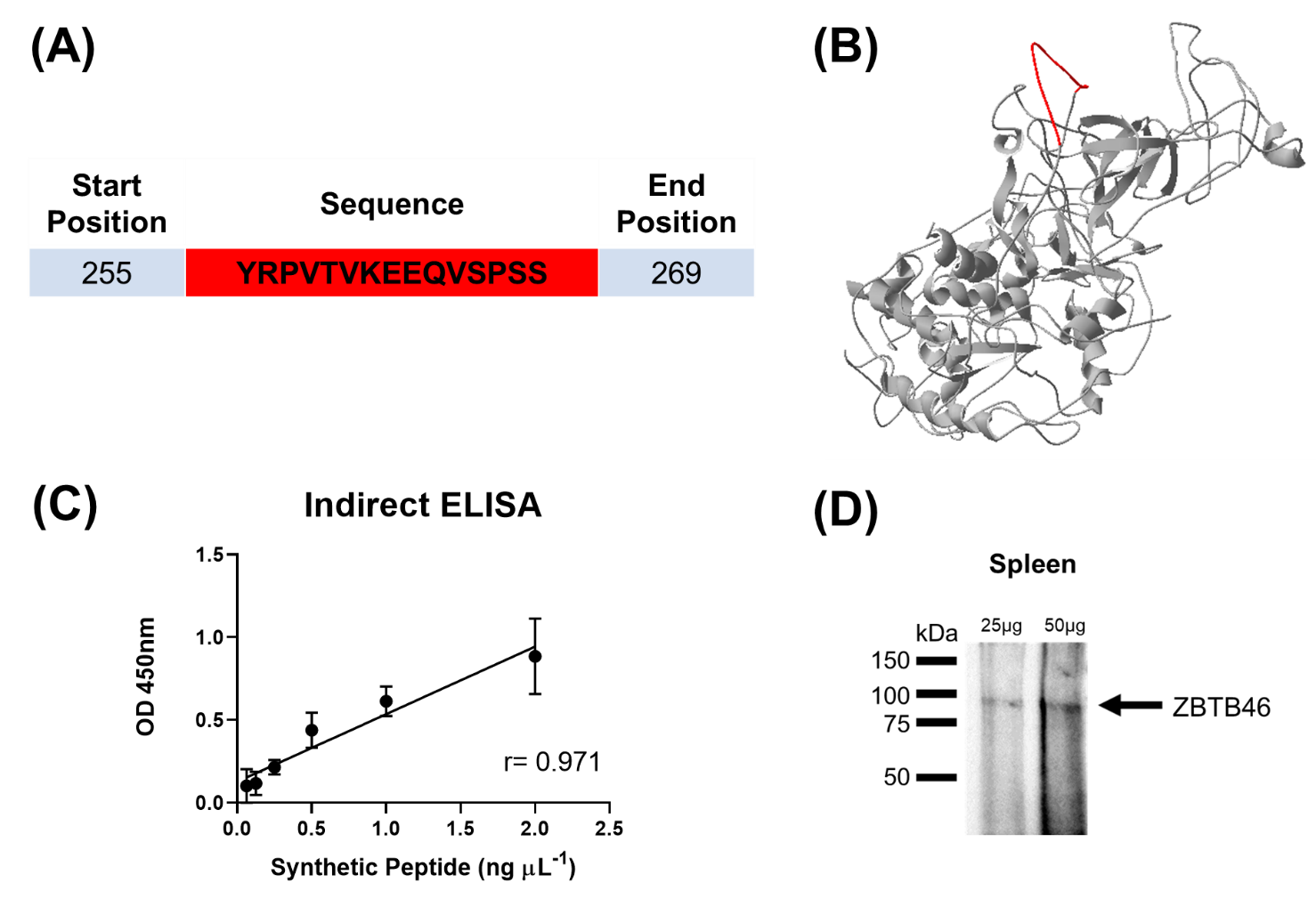


**Supplementary Figure 1.** The antibody against Zinc Finger and BTB Domain Containing 46 (zbtb46) was obtained from mouse using a synthetic peptide as antigen. (A): in red, antigenic peptide selected from A0A1S3LH90 (UniProtKB). (B): three-dimensional model prediction by Phyre2. In red: antigenic peptide. (C) Affinity test against synthetic peptide by indirect ELISA at 450 nm. (D) The specificity to recognize zbtb46 was evaluated by Western blotting using a pool of six spleen samples from FM-fish group. The arrows indicate the band corresponding to the expected zbtb46 molecular weight (88 kDa).
